# Modeling the indirect economic implications of musculoskeletal disorders and treatment

**DOI:** 10.1186/1478-7547-11-5

**Published:** 2013-03-15

**Authors:** Timothy M Dall, Paul Gallo, Lane Koenig, Qian Gu, David Ruiz

**Affiliations:** 1 IHS Global Inc., 1150 Connecticut Ave., NW, Suite 401, Washington, DC, 20036, USA; 2 KNG Health Consulting, 1445 Research Blvd., Suite 320, Rockville, MD, 20850, USA

## Abstract

**Background:**

Musculoskeletal disorders impose a substantial economic burden on American society, but few studies have examined the economic benefits associated with treating such disorders. The purpose of this research is to estimate the indirect economic implications of activity limitations associated with musculoskeletal disorders and to quantifying the potential economic gains from elective surgery to treat arthritis of the knee and hip.

**Methods:**

Using regression analysis with the National Health Interview Survey (2004-2010 data, n=185,829 adults) we quantify the relationship between severity of activity limitations (walking, sitting, standing, etc.) and employment, household income, missed work days, and receipt of supplemental security income for disability. Activity limitations are combined to create an index similar to the Functional Ability Index from the Short Form 36 Health Questionnaire (SF-36) often used in clinical trials to measure patient functional mobility. This index is included in the regression analyses. We use data from published, prospective clinical trials to establish the improvement in patient functional ability following surgery to treat arthritis of the knee and hip.

**Results:**

Improved physical function is associated with higher likelihood of employment, higher household income and fewer missed work days for those who are employed, and reduced likelihood of receiving supplemental security income for disability. The magnitude of the impact and statistical significance vary by activity limitation and severity. Each percentage point increase in the index value is associated with a 2-percentage-point increase in the odds of being employed, a 3-percentage-point-day decline in work days missed and an additional $180 in annual household income if employed, and a 2-percentage-point decline in the odds of receiving supplemental security income for disability. All estimates are statistically significant at the 0.05 level.

**Conclusions:**

Using a large, representative sample of non-institutionalized adults in the U.S., we find that physical activity limitations are associated with worse economic outcomes across multiple economic metrics. Combined with estimates of improved functional ability following knee and hip surgery, we quantify some of the economic benefits of surgery for arthritis of the knee and hip. This information helps improve understanding of the societal benefits of medical treatment for musculoskeletal conditions.

## Introduction

Musculoskeletal (MSK) disorders impose a substantial burden on American society, with national estimates of MSK burden in 2004 of $510 billion in direct medical expenditures and $339 billion in lost productivity [1]. The high prevalence of MSK disorders, with many people simultaneously experiencing multiple disorders, includes 61.6 million with chronic joint pain, 62 million with low back pain, and 31.4 million with neck pain [1]. Arthritis is a major cause of joint pain, and an estimated 51.2 million adults in 2008 suffered from arthritis. Osteoarthritis alone affects nearly 27 million U.S. adults and is the fifth leading cause of disability in the elderly [2].

MSK disorders cause pain, loss of physical function, and decline in mental health, all of which adversely affect a person’s ability to pursue gainful employment [3-5]. A study of retirement among 14,474 construction workers in the U.S. found that after controlling for demographics and presence of chronic medical conditions, each point decrease in physical functioning was associated with a 6% increase in the likelihood of retiring the following year [6]. Among construction workers engaged in roofing, those with MSK disorders were eight times more likely to leave their occupation than their peers with no MSK disorders. Studies from several European countries (which often have disease registries that allow one to track employment status by presence of chronic conditions) find that increasing severity of MSK disorders increases the propensity of workers to retire earlier [7-9]. A study of workers aged 50 to 65 in the United Kingdom reports that after controlling for demographics, economic well-being, and various measures of health status, a person’s reported difficulty walking a quarter-mile, especially when symptoms included lower limb pain and/or shortness of breath, was predictive of early work exit (odds ratio=2.23) [7].

For employed adults, the presence of MSK-related conditions can increase the number of work days missed (or absenteeism). Puolakka et al. analyzed data for 152 gainfully employed patients undergoing surgery for lumbar disc herniation who were evaluated for back-related loss of working time [10]. Of all patients, 53% reported musculoskeletal-related sick leave or a work disability pension and 10% were awarded a permanent work disability pension due to back pain. According to the Bureau of Labor Statistics, (as cited in the National Research Council and Institute of Medicine’s *Panel on Musculoskeletal Disorders and the Workplace Commission on Behavioral and Social Sciences and Education*) nearly one million people each year report taking time away from work to treat and recover from musculoskeletal pain or loss of function due to overexertion or repetitive motion either in the low back or upper extremities [11]. The Bureau of Labor Statistics reports that in 2011 there were approximately 387,800 workers who missed work (median days absent =11) because of occupational MSK disorders [12].

While many studies have quantified the burden of musculoskeletal disorders and cost-effectiveness of treatment [13-17], few studies address the economic value of services provided to treat these disorders. Mobasheri et al. examined the employment status of hip-replacement patients in the United Kingdom and found that of 81 total hip patients, nearly all who were working preoperatively returned to employment following surgery, and nearly half of those not working pre-operatively due to hip pain regained employment postoperatively [3]. Hip and knee joint replacement surgery is reserved for patients with late- and/or end-stage osteoarthritis. Pain management and other non-operative treatments can postpone joint replacement surgery. However, osteoarthritis is a chronic and progressive condition. At the point of end-stage disease, where bone meets bone, knee and hip replacement is the most effective treatment for relieving pain and improving function.

From the perspectives of the patient, employers, and society, the value of appropriate medical treatment extends beyond current and future medical expenditures. Value includes: whether a person could remain productively employed, the avoidance of payments for disability or long term care, the avoidance of expenditures related to reduced mobility (e.g., home modifications), and overall improved quality of life.

The paucity of information on these indirect economic implications of treatment for patients in the U.S. stems in large part from the lack of longitudinal data that tracks patient outcomes over time (pre- and post-treatment) and relates outcomes to economic activities such as labor force participation and non-medical expenditures. Longitudinal studies of patients in other countries (Canada and Spain) have quantified decreases in pain, increases in physical functionality, and improvements in quality of life following elective hip and knee replacement associated with arthritis [18-21]. Still, these studies have not collected information on or attempted to link treatment outcomes to patients’ economic activities. Information on the indirect economic implications of treatment, combined with information on direct medical cost implications of treatment and improved quality of life, is needed to understand the total value of treatment so that society can more efficiently allocate scarce resources [22].

This paper introduces a methodological approach to infer the indirect economic benefits of interventions to improve physical functions when direct data do not exist. We apply this approach to quantifying the potential economic gains from elective surgery to treat arthritis of the knee and hip, but the method could be applied to other interventions. Such information will provide a more complete picture of the burden of MSK disorders and the benefits of treatment in the absence of lengthy and expensive randomized clinical trials that would be needed to provide definitive information on the short-and-long term economic implications of treatment.

## Methods

To estimate indirect costs by severity of MSK disorder we first estimate the relationship between patient functional limitations and indirect cost factors: employment, work days missed, household income, and disability payments. After establishing this relationship, we combine published information from clinical trials that quantify changes in patient functional ability following elective surgery for arthritis of the hip and knee. The impact of surgery on functional ability, combined with the estimated relationship between functional ability and indirect cost factors, provides estimates of the indirect economic benefits from total hip and total knee replacements.

### Estimating the relationship between functional limitations and economic factors

Our review identified no data sources in the U.S. that directly link treatment for MSK disorders to patients’ economic activity (such as ability to remain employed). However, the National Health Interview Survey (NHIS), which is sponsored by the Centers for Disease Control and Prevention, collects information from a stratified random sample of the U.S. population on physical function, economic factors such as employment status and income, and other patient characteristics [23]. Our analysis combined the 2004 through 2010 NHIS files to increase the sample size, resulting in a sample of 185,829 adults age 18 and older living in non-institutional settings. The NHIS asks respondents: *By yourself, and without using any special equipment, how difficult is it for you to…*

•*Walk a quarter of a mile - about 3 city blocks?*

•*Walk up 10 steps without resting?*

•*Sit for about 2 hours?*

•*Reach up over your head?*

•*Stand or be on your feet for about 2 hours?*

•*Stoop, bend, or kneel?*

•*Lift or carry something as heavy as 10 pounds such as a full bag of groceries?*

•*Push or pull large objects like a living room chair?*

Responses to each question include: (1) Not at all difficult, (2) Only a little difficult, (3) Somewhat difficult, (4) Very difficult, (5) Can't do at all. Our analysis focuses only on activity limitations where the person indicates that back pain, bone/joint injury, or arthritis contributed to his/her limitations.

Using regression analysis we compare economic outcomes for adults with activity limitations to economic outcomes for adults without activity limitations—controlling for age group (18–39, 40–44, 45–49, 50–54, 55–59, 60 to 64, 65–69, and 70 years and over), sex, highest education attainment (high school diploma, baccalaureate degree, post-baccalaureate degree), and occupation (for analysis of the employed population).

We used two approaches to measure level of physical functioning. First, responses to physical functioning questions were decoupled such that for each question, each response (e.g., “only a little difficult,” “somewhat difficult,” “very difficult,” “can't do at all”, and “not at all difficult”) a binary variable (1=yes, 0=no) was created. Persons claiming that the physical functioning task was “not at all difficult” were used as the comparison group. For the second approach (described later) we created a Physical Function Index variable that combines multiple physical function variables. The first approach allows us to validate the relationships because we see an inverse relationship between increasing level of difficulty and declining economic activity. The second approach allows us to combine the NHIS analysis with findings in the published literature.

Logistic regression was used to quantify the effect of patient activity limitations on employment probability and probability of receiving supplemental security income (SSI) for disability. Ordinary least squares regression was used to quantify the impact of activity limitations on household income for the employed population. Household income in the NHIS is reported in one of eight ranges (rather than as a continuous variable). For use in the regression, we convert the range to a semi-continuous variable using the midpoint of the range in which a household lies as a proxy for that household’s income. Data from 2004 through 2009 are adjusted to 2010 dollars using the consumer price index.

To analyze missed work days we used a negative binomial regression rather than Poisson regression (which typically is used for count data) because of over dispersion with the work days missed variable. For the 0.5% of the employed population who report more than 100 missed work days per year, we cap missed work days at 100. This reduces the problem with over dispersion of the missed work days variable, but also reflects that long work absences are counted as disability rather than absenteeism. Most employer policies will move an employee from short-term to long-term disability status after approximately three to size months (with the midpoint of this range being approximately 100 work days). Disability is modeled separate from absenteeism.

### Estimating the relationship between surgery and improvement in functionality

We identified three published clinical trials that report change in patient physical function pre-and-post treatment for elective knee and hip replacement for arthritis (Table 1) [18,19,21]. Each of the three studies used the Short Form (SF) 36 Health Questionnaire to collect patient functional ability both pre-treatment and six months following treatment [24]. Two studies report information related to total hip replacement [THR], while all three report information for total knee replacement [TKR]). Two studies are based on a population in Canada; the third is based on a population in Spain. All three studies report similar findings on patient physical functioning.

**Table 1:** Observed Improvement in SF-36 physical function scores

**Study**	**Treatment**	**Pre-intervention score**	**Improvement at 6 months**	**Population**
		**(Sample size)**	**(Sample size)**	
Quintana et al. [21]	THR	17.98	+34.44	Consecutive patients with diagnoses of osteoarthritis on waiting lists to undergo THR or TKR at 7 teaching hospitals in the Spanish National Health Service; patient medical records indicate THR/TKR was appropriate treatment; excludes patients with severe morbidity such as cancer or terminal condition.
		(N=575)	(N=434)	
	TKR	19.49	+25.79	
		(N=557)	(N=414)	
Jones et al. [19]	TKR	21.0	+23.8	Prospective, longitudinal study of an inception cohort of surgical candidates in a Canadian health care region (Capital Health) between Feb 1996 and Feb 1998.
		(N=276)	(N=273)	
Jones et al. [18]	THR	20	+30	Prospective, community-based cohort recommended for THR or TKR within a Canadian health care region (Edmonton, Alberta) between Dec 1995 and Jan 1997.
	(age <80)	(N=163)	(N=163)	
	THR	13	+26	
	(age ≥80)	(N=34)	(N=34)	
	TKR	21	+26	
	(age <80)	(N=221)	(N=221)	
	TKR	17	+18	
	(age ≥80)	(N=35)	(N=36)	

Notes: THR (total hip replacement), TKR (total knee replacement).

A challenge when combining estimates of the relationship between activity limitations and economic outcomes (from the NHIS regression analysis) with estimates of improvement in functional ability (using SF-36 results from published trials) is that one cannot precisely recreate the SF-36 Functional Ability Index from NHIS data. Still, the functional ability questions in the NHIS we think are sufficiently similar that one can create a proxy index for functional ability similar to that created using the SF-36. Table 2 shows how the NHIS functional ability questions map to the SF-36 functional ability questions.

**Table 2:** Comparison of physical function questions

**SF-36 physical function area**	**National health interview survey**
**(1–3 points each)**	
Moderate activities, such as moving a table, pushing a vacuum cleaner,bowling, or playing golf	Push or pull large objects like a living room chair (1–3 points)
Lifting or carrying groceries	Lift or carry a 10-pound bag (1–3 points)
Climbing several flights of stairs	Walk up steps without resting (2–6 points)
Climbing one flight of stairs	
Bending, kneeling, or stooping	Stoop, bend, or kneel (1–3 points)
Walking more than a mile	Walk a quarter of a mile (3–9 points)
Walking several blocks	
Walking one block	
Vigorous activities, such as running, lifting heavy objects, participating in strenuous sports	**No corresponding variable in NHIS**
Bathing or dressing yourself	

Note: for the SF-36 total points = 30 and the patient’s Functionality Index = (∑points)/30. For the NHIS total points = 24 and the patient’s Index = (∑points)/24.

The SF-36 Functional Ability Index value for someone with no functional limitations is 100%, whereas someone with limitations will have a value below 100%. For each of the ten SF-36 questions used to assess physical functionality, the SF-36 Functional Ability Index assigns three points if the person is “not limited at all,” two points if the person is “limited a little,” and one point if the person is “limited a lot.” The sum of all the points is then divided by 30 to create the index value for that person. Of the ten SF-36 questions used to assess physical functionality, eight are related to the NHIS questions (Table 2). One difference, though, is that there are only three possible responses to the SF-36 (whereas there are five responses to the NHIS). When creating our index to use in the regression equations, similar to the SF-36, we assign a person three points if they respond “not at all difficult.” A person is given two points if in the NHIS they respond “only a little difficult” or “somewhat difficult,” and one point if they respond “very difficult” or “can't do at all.” The SF-36 index weights all functional areas equally. When creating a proxy index with the NHIS, though, we give double weight to the “walk up steps without resting” measure and triple weight to the “walk a quarter of a mile” variable because they map into, respectively, two and three SF-36 measures. For the NHIS Physical Function Index, a person receives 24 points if they have no limitations, so we divide each person’s total points by 24 to create the index.

The physical function index does not include the questions for mobility limitations related to sitting, reaching, and standing. We include these mobility-related questions in the regression with the physical function index (Table 2) for consistency with the regression that uses the full set of physical function variables (Table 1). Patient responses to limitations related to sitting, reaching and standing are correlated with the physical function index, and including these additional mobility questions produces more conservative estimates of the economic benefits of improving mobility (as reflected solely by the physical function index).

## Results

The NHIS population analyzed covers the years 2004–2010, and includes 185,829 adults aged 18 and older. Of these, 45% were male, 53% were employed, and 4% received SSI for disability. Among the employed, the average annual work days lost was 3.8 days. Among the population analyzed, some reported at least some difficulty walking a quarter mile (9.6%); walking up 10 steps without resting (7.8%); sitting for about two hours (7.1%); reaching above their head (5.7%); standing or being on their feet for about two hours (11.7%); stooping, bending, or kneeling (14.5%); lifting or carrying something as heavy as 10 pounds (6.8%); and pushing or pulling large objects like a living room chair (8.95%).

Increased employment and productivity associated with TKR and THR accrue primarily to patients who otherwise would have been in the workforce. The Agency for Healthcare Research and Quality (AHRQ) reports that in the 2010 Nationwide Inpatient Sample there were 721,443 hospitalizations associated with TKR and 453,663 hospitalizations associated with hip replacement (though this number includes both partial and total hip replacement) [25]. Approximately 44 percent of TKR patients were under age 65, and 39 percent of patients receiving hip replacement were under age 65.

TKR and THR are expensive procedures—with expenses including hospital charges, physician fees, and implant costs. Lavernia et al. report that in 2007 the average cost to Medicare for total hip arthroplasty was approximately $10,500 for hospital payments, $6,400 for implants, and $1,300 for physician payments [26]. A recent study on knee replacement (covering 19,000 Medicare surgeries and 32,000 surgeries covered by commercial insurers) found that during the 180 days surrounding the surgery Medicare paid an average of $22,611 per patient in 2011 while commercial insurers paid an average of $25,872 [27].

### Economic burden associated with functional limitations

The regression results (Tables 3 and 4) suggest that decreased physical function reduces the likelihood of employment, reduces household income and increases missed work days for those who are employed, and increases the likelihood of receiving supplemental security income for disability. The magnitude of the impact and statistical significance vary by activity limitation and severity. Relative to a person with no difficulty walking a quarter-mile without special equipment, for example, the odds of being employed falls by 16% if the person has only a little difficulty walking, by 24% if somewhat difficult to walk, by 32% if very difficult to walk, and by 44% if unable to walk (Table 1).

**Table 3:** Regression results from national health interview survey

	**All adults (age 18+)**		**Employed adults (age 18-74)**	
	**Employed**^**(1)**^	**Receives SSI**^**(1)**^	**Household income ($)**^**(2)**^	**Missed work days**^**(3)**^
**Intercept**			22,242*	
**Male**	1.73*	0.89*	2,141*	0.74*
**Age 40-44 vs. <40**	1.51*	1.46*	7,192*	1.12*
**Age 45-49 vs. <40**	1.55*	1.70*	7,887*	1.10*
**Age 50-54 vs. <40**	1.40*	1.93*	7,917*	1.10*
**Age 55-59 vs. <40**	0.95*	2.13*	7,383*	1.17*
**Age 60-64 vs. <40**	0.45*	2.06*	5,940*	1.11*
**Age 65-69 vs. <40**	0.16*	1.59*	3,908*	1.07
**Age 70+ vs. <40**	0.05*	0.76*	(1,804)*	0.84*
**Difficulty walking- vs. no difficulty**				
**Only a little difficult**	0.84*	1.36*	(1,790)*	1.26*
**Somewhat difficult**	0.77*	1.49*	(3,434)*	1.40*
**Very difficult**	0.68*	1.15	(3,852)*	1.51*
**Can't do at all**	0.57*	1.19	(4,347)*	1.54*
**Difficulty climbing- vs. no difficulty**				
**Only a little difficult**	1.01	1.51*	(2,844)*	1.08
**Somewhat difficult**	0.94	1.78*	(3,102)*	1.10
**Very difficult**	0.98	2.08*	(2,551)*	1.26*
**Can't do at all**	0.78*	2.23*	(1,663)	1.35
**Difficulty sitting-vs. no difficulty**				
**Only a little difficult**	0.97	0.97	769	1.07
**Somewhat difficult**	0.81*	1.24*	450	1.14*
**Very difficult**	0.77*	1.38*	1,846	1.16
**Can't do at all**	0.53*	1.29*	(1,895)	1.08
**Difficulty reaching-vs. no difficulty**				
**Only a little difficult**	0.91*	0.92	201	1.07
**Somewhat difficult**	0.74*	1.00	523	1.18*
**Very difficult**	0.61*	1.23*	(896)	1.11
**Can't do at all**	0.55*	1.09		1.76*
**Difficulty standing-vs. no difficulty**				
**Only a little difficult**	0.78*	1.24*	974	1.01
**Somewhat difficult**	0.63*	1.32*	346	1.34*
**Very difficult**	0.48*	1.65*	1,491	1.18*
**Can't do at all**	0.31*	1.78*	(212)	1.26*
**Difficulty stooping- vs. no difficulty**				
**Only a little difficult**	1.10*	0.82*	731	1.09
**Somewhat difficult**	1.08*	0.77*	(347)	1.19*
**Very difficult**	1.10	0.79*	(737)	1.16*
**Can't do at all**	1.09	0.68*	(357)	1.42*
**Difficulty carrying- vs. no difficulty**				
**Only a little difficult**	0.77*	1.60*	(1,224)	0.92
**Somewhat difficult**	0.79*	1.61*	(2,497)*	0.94
**Very difficult**	0.55*	2.31*	(5,448)*	0.89
**Can't do at all**	0.64*	2.15*	(4,122)	1.26
**Difficulty pushing- vs. no difficulty**				
**Only a little difficult**	0.89*	1.04	(62)	1.15*
**Somewhat difficult**	0.90*	1.12	(1,164)	1.55*
**Very difficult**	0.81*	1.25*	(933)*	1.43*
**Can't do at all**	0.56*	1.18	935	2.08*
**Has mobility difficulty due to**				
**Back pain**	1.09*	1.01	(488)	1.35*
**Joint injury**	1.09*	0.95	604	2.02*
**Musculoskeletal condition**	0.86*	1.20*	(835)	1.69*
**Arthritis**	1.16*	1.11	42	1.16*
**Year 2004 vs. 2010**	1.25*	0.86*	2,305*	1.04
**Year 2005 vs. 2010**	1.22*	0.87*	2,393*	1.12*
**Year 2006 vs. 2010**	1.20*	0.85*	2,328*	1.07*
**Year 2007 vs. 2010**	1.18*	0.95*	(2,583)*	1.09*
**Year 2008 vs. 2010**	1.13*	0.98*	2,166*	1.04
**Year 2009 vs. 2010**	1.03	0.93*	559	1.02
**Highest educational attainment**				
**High school degree**	1.45*	0.43*	18,214*	1.11*
**College (baccalaureate) degree**	1.93*	0.24*	37,781*	1.02
**Post baccalaureate degree**	1.95*	0.20*	49,648*	0.97
**Occupation**				
**Management**			8,981*	1.22*
**Finance**			7,650*	1.37*
**Computer**			8,524*	1.57*
**Architect**			9,349*	1.50*
**Social Sciences**			1,878	1.38*
**Social Services**			(4,019)*	1.74*
**Legal**			7,838*	1.55*
**Educate**			(1,624)*	1.21*
**Arts**			318	1.28*
**Health practitioner**			7,439*	1.35*
**Healthcare support**			(3,530)*	1.49*
**Protective services**			5,146*	2.00*
**Food preparation**			(6,799)*	1.26*
**Cleaning**			(5,226)*	1.40*
**Personal care**			(3,958)*	1.16*
**Sales**			2,187*	1.31*
**Administrative support**			2,442*	1.52*
**Farm**			(2,767)*	1.33*
**Construction**			330	1.66*
**Maintenance**			4,851*	1.82*
**Production**			679	1.68*
**Transport**			(215)	1.81*
**Military**			11,432*	0.94
**Summary Statistics**	N=185,813	N=185,813	N=101,098	N=115,752
	Wald=31,794*	Wald=6,043*	R-sq=0.265	Pearson Chi-sq =241,514

Notes: (1) Odds ratios from Logistic regression. (2) Coefficients from Ordinary Least Squares regression. Comparison group is female, under Age 40, no activity limitations, year 2010, unemployed or occupation not available (for regressions that include occupation), and no high school degree. (3) Rate ratios from negative binomial regression. Comparison group is same as (2) above. * Statistically significant at the 0.05 level.

**Table 4:** Regression results from national health interview survey (Functionality index)

	**All adults (age 18+)**		**Employed adults (age 18-74)**	
	**Employed**^**(1)**^	**Receives SSI**^**(1)**^	**Household income ($)**^**(2)**^	**Missed work days**^**(3)**^
**Intercept**			4,231	
**Male**	1.75*	0.86*	2,175*	0.73*
**Age 40-44 vs. <40**	1.51*	1.47*	7,198*	1.12*
**Age 45-49 vs. <40**	1.54*	1.71*	7,869*	1.10*
**Age 50-54 vs. <40**	1.39*	1.95*	7,889*	1.10*
**Age 55-59 vs. <40**	0.95*	2.15*	7,336*	1.18*
**Age 60-64 vs. <40**	0.45*	2.08*	5,851*	1.11
**Age 65-69 vs. <40**	0.16*	1.58*	3,709*	1.06*
**Age 70+ vs. <40**	0.05*	0.77*	(1,810) *	0.84*
**Physical Function Index**	1.02*	0.98*	180*	0.97*
**Difficulty sitting-vs. no difficulty**				
**Only a little difficult**	0.94	1.06	87	1.10
**Somewhat difficult**	0.81*	1.32*	189	1.14*
**Very difficult**	0.72*	1.57*	1,278	1.23*
**Can't do at all**	0.50*	1.35*	(1,323)	1.19
**Difficulty reaching-vs. no difficulty**				
**Only a little difficult**	0.84*	1.09	(361)	1.08
**Somewhat difficult**	0.69*	1.21*	37	1.19*
**Very difficult**	0.53*	1.55*	(1,093)	1.11
**Can't do at all**	0.46*	1.18		2.01*
**Difficulty standing-vs. no difficulty**				
**Only a little difficult**	0.75*	1.47*	(116)	1.06
**Somewhat difficult**	0.64*	1.65*	(870)	1.40*
**Very difficult**	0.47*	2.27*	(574)	1.23*
**Can't do at all**	0.29*	2.05*	(337)	1.28*
**Has mobility difficulty due to**				
**Back pain**	1.09*	1.02	(2,116)*	1.50*
**Joint injury**	1.14*	0.94	(1,185)	2.19*
**Musculoskeletal condition**	0.86*	1.21*	(834)	1.69*
**Arthritis**	1.20*	1.15*	(1,893)*	1.28*
**Year 2004 vs. 2010**	1.24*	0.86	2,302*	1.04
**Year 2005 vs. 2010**	1.22*	0.87*	2,362*	1.12*
**Year 2006 vs. 2010**	1.20*	0.85*	2,280*	1.07*
**Year 2007 vs. 2010**	1.18*	0.95	(2,616)*	1.09*
**Year 2008 vs. 2010**	1.13*	0.97	2,169*	1.04
**Year 2009 vs. 2010**	1.04*	0.93	557	1.02
**Highest educational attainment (comparison is no HS degree)**				
**High school degree**	1.45*	0.42*	18,262*	1.11*
**College (baccalaureate) degree**	1.93*	0.24*	37,808*	1.02
**Post baccalaureate degree**	1.96*	0.19*	49,722*	0.97
**Occupation (comparison is occupation unknown)**				
**Management**			9,017*	1.22*
**Finance**			7,635*	1.36*
**Computer**			8,535*	1.56*
**Architect**			9,342*	1.49*
**Social Sciences**			1,828	1.38*
**Social Services**			(4,099)*	1.74*
**Legal**			7,877*	1.54*
**Educate**			(1,591)*	1.21*
**Arts**			318	1.28*
**Health practitioner**			7,404*	1.35*
**Healthcare support**			(3,600)*	1.50*
**Protective services**			5,142*	1.99*
**Food preparation**			(6,835)*	1.26*
**Cleaning**			(5,245)*	1.39*
**Personal care**			(4,045)*	1.16*
**Sales**			2,178*	1.30*
**Administrative support**			2,420*	1.52*
**Farm**			(2,843)*	1.34*
**Construction**			342	1.66*
**Maintenance**			4,799*	1.82*
**Production**			638	1.67*
**Transport**			(287)	1.80*
**Military**			11,409*	0.93
**Summary Statistics**	N=185,829	N=185,829	N=100,471	N=115,752
	Wald=31,831*	Wald=5,718*	R-sq=0.265	Pearson Chi-sq =241,078

Notes: (1) Odds ratios from Logistic regression. (2) Coefficients from Ordinary Least Squares regression. Comparison group is female, under age 40, no activity limitations, year 2010, unemployed or occupation not available (for regressions that include occupation), and no high school degree. (3) Rate ratios from negative binomial regression. Comparison group is same as (2) above. * Statistically significant at the 0.05 level.

**Figure 1:**
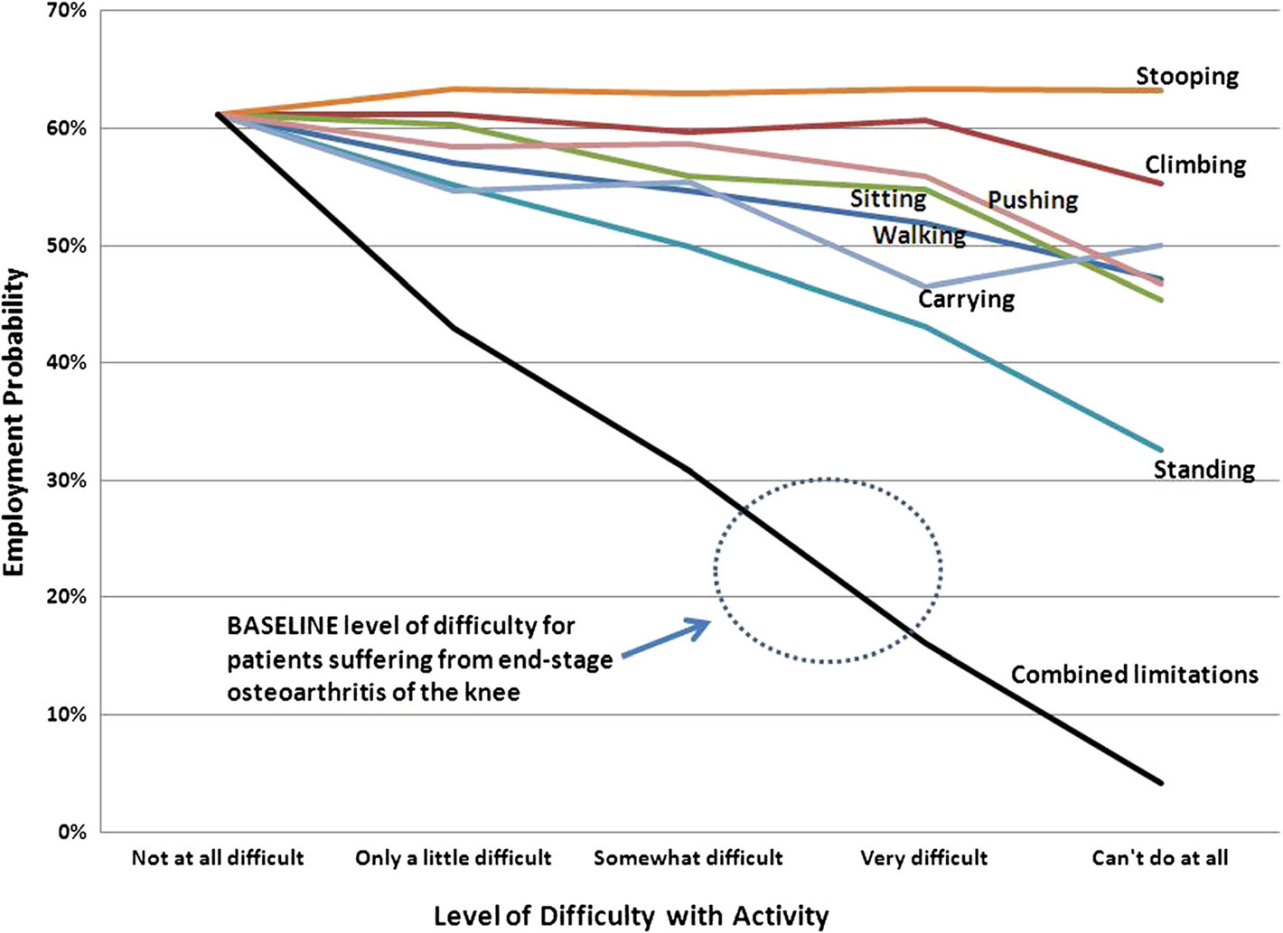
**Predicted Probability of Employment: Male Age 60 to 64 Years.** Increased difficulty performing physical activities is associated with a decline in probably if being employed, controlling for demographics and other determinants (e.g., education level) of employment probability.

Because the underlying probability of employment varies by age, sex, education level, and other factors, the economic implications of MSK limitations will vary by patient. For a 60-to-64-year-old male who has no difficulty performing the activities described previously (walking, standing, etc.) the probability of being employed in 2010 was 61%. A little difficulty walking a quarter-mile without special equipment drops the probability to 57%, and this probability falls to 55%, 52%, and 47%, respectively, if somewhat difficult, very difficult, or unable to walk (Figure 1). Areas where physical difficulties are associated with substantial and statistically significant declines in employment probability are walking, sitting, standing, and carrying. The employment impact of difficulties with climbing, stooping, and pushing appear to be modest and not statistically significant in most cases (though the economic impact of MSK-related disabilities can vary substantially by type of employment).

Patients with MSK disorders will often experience all or most of these activity limitations simultaneously. The line labeled “Combined limitations” shows the predicted employment probability if the person has difficulty with all the activities analyzed. Regarding candidates for elective knee replacement due to arthritis, the consensus of four orthopaedic surgeons who specialize in TKR suggests that pre-surgery patients will find it somewhat-to-very difficult to walk a quarter-mile; walk up 10 steps without resting; sit for two hours; stand for two hours; stoop, bend, or kneel; lift or carry 10 pounds; or push or pull large objects. While the national average probability of working is 61% for a 60 to 64 year old male without these limitations, our analysis suggests that a similar male who had the same functional limitations as a person requiring hip replacement would have only a 21-25% probability of being employed.

**Figure 2:**
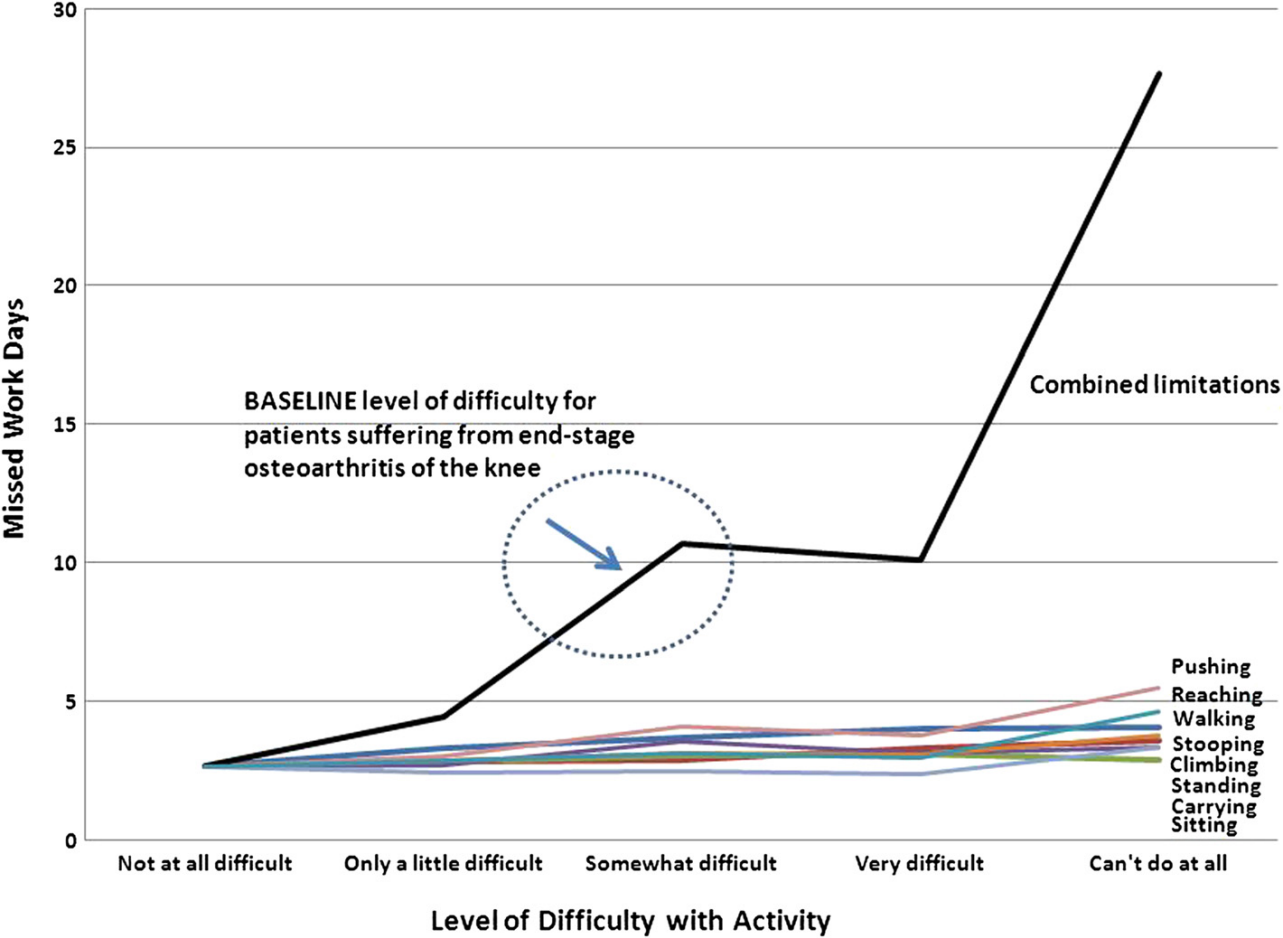
**Predicted Missed Work Days: Male Age 60 to 64 Years.** Increased difficulty performing physical activities is associated with higher number of missed work days (absenteeism) among the employed population, controlling for demographics and other determinants (e.g., occupation, education level) of missed work days.

Increasing levels of difficulty performing physical activities is associated with higher work days missed (Figure 2). Using the physician consensus estimates of patients activity limitations pre-surgery, we calculate that for a male age 60 to 64 with the same activity limitations as a person requiring elective TKR the annual missed work days is 9–10 (versus 2–3 missed work days for someone without these activity limitations). Missed work days vary slightly by occupation, are higher for high school graduates versus college graduates, and have declined from 2005 through 2010.

**Figure 3:**
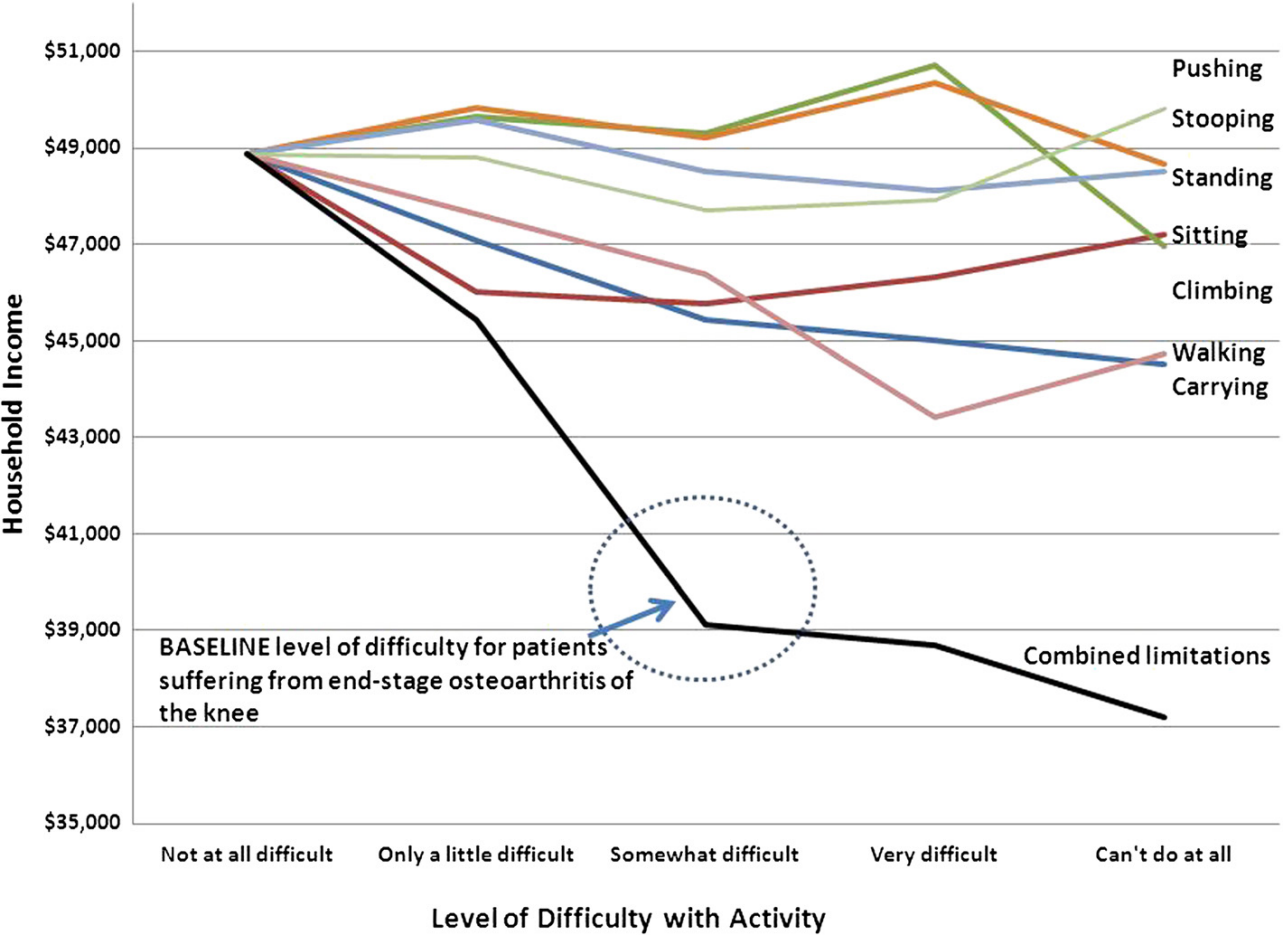
**Predicted Household Income: Employed Male Age 60 to 64 Years.** Increased difficulty performing physical activities is associated with a decline in household income among the employed population, controlling for demographics and other determinants (e.g., occupation, education level) of household income.

Among the employed, having difficulty carrying, walking and climbing is associated with a decrease in annual household income (Figure 3). Under the combination scenario, an employed male age 60 to 64 years with the activity limitations of a person requiring TKR is predicted to reduce annual income by approximately $9,500 relative to a similarly employed person without any of these limitations. Income level varies substantially by occupation, education level, and demographics consistent with published findings.

**Figure 4:**
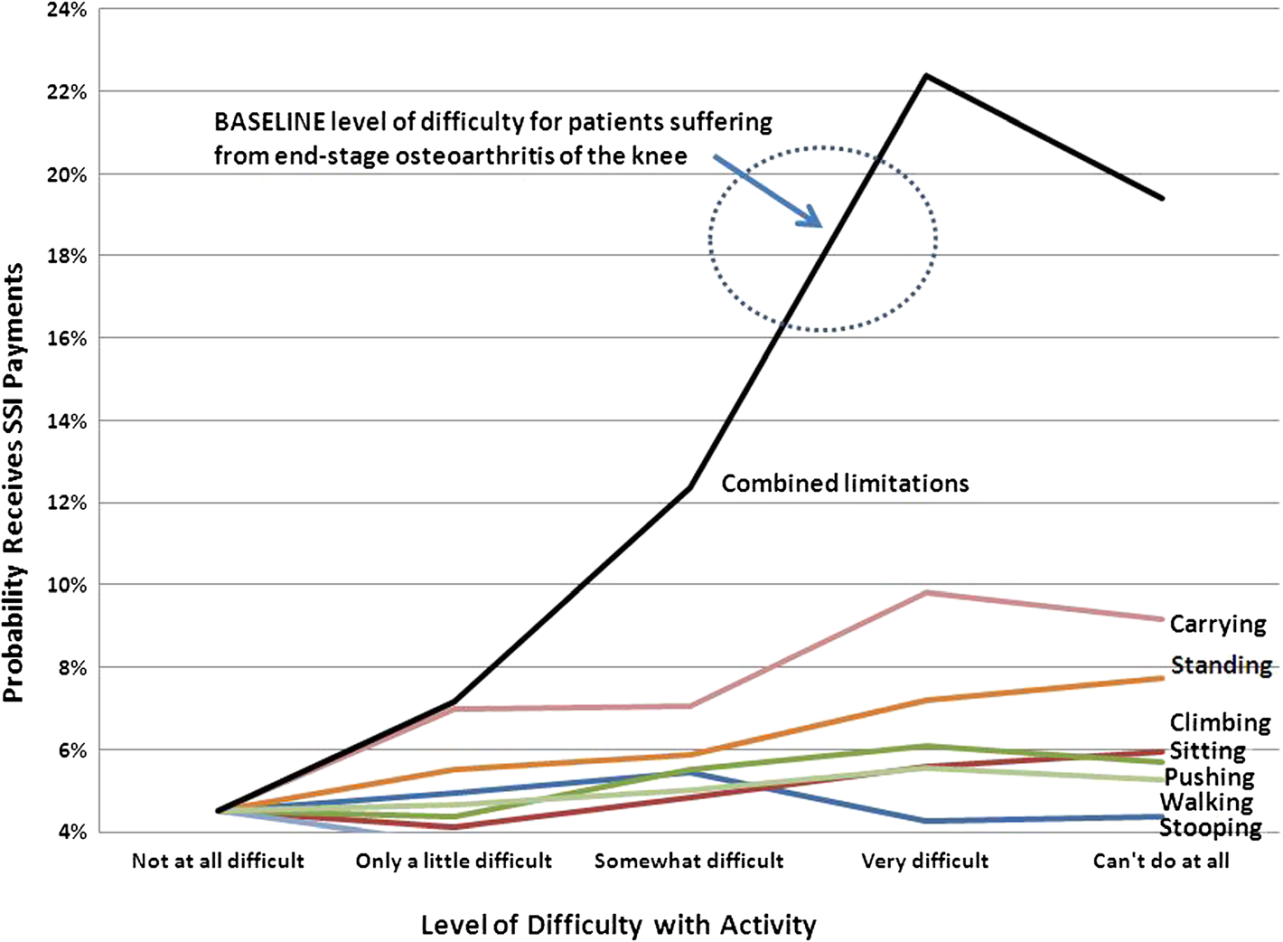
**Predicted Probability Receiving SSI Payments for Disability: Male Age 60 to 64 Years.** Increased difficulty performing physical activities is associated with higher probability of receiving supplemental security income payments for disability, controlling for demographics and other person characteristics (e.g., education level).

For a male age 60 to 64, if there were no MSK-related activity limitations then the probability of receiving Supplemental Security Income (SSI) payments for disability is 4.5% (Figure 4). The estimated probability of receiving SSI would be about 16-20% if his activity limitations were similar to that of a candidate for elective TKR.

### Potential economic benefits associated with treatment

When using the calculated Physical Function index as an explanatory variable in the NHIS regressions, each 1-percentage-point increase in the index value is associated with a 2.3 percentage point increase in the odds of being employed, a 3-percentage-point decline in rate of work days missed (if employed), an additional $180 in annual household income, and a 2-percentage-point decline in the odds of receiving supplemental security income for disability. All estimates are statistically significant at the 0.05 level. For the “Combination” scenario that reflects consensus from the physician panel regarding the level of difficulty likely experienced by patients who are candidates for TKR, the predicted economic outcomes are relatively similar regardless of whether using the index variable in the regressions or entering dichotomous variables for each separate activity. For a person with activity limitations similar to a candidate for TKR, the estimated employment probability is 23.2% when using the dichotomous variables and 17.0% when using the index variable.

Summary estimates of the economic activity impact of TKR and THR—based on our regression results combined with improvement in SF-36 Physical Function Index— tend to be consistent across the three studies analyzed (Table 5). For a male age 60 to 64 who undergoes TKR, for example, the improvement in SF-36 functionality score is consistent with 20 to 21 percentage point higher probability of being employed, $4,300 to $4,700 increase in annual household income and 6 fewer missed work days for those who are employed, and a decline in probability of receiving SSI payments for disability of about 17 percentage points.

**Table 5:** Estimated economic benefits of THR and TKA: male patient age 60 to 64

**Source of treatment impact on physical function**	**Percentage point increase in employment probability**	**Change in annual household income**	**Change in missed work days**	**Change in probability of receiving supplemental security income payments**
***Total knee replacement***				
Quintana et al. [21]	20.6%	$4,600	−6.4	−17.2%
Jones et al. [19]	20.1%	$4,300	−5.9	−16.6%
Jones et al. [18]	21.1%	$4,700	−6.1	−16.8%
***Total hip replacement***				
Quintana et al. [21]	28.4%	$6,200	−21.8	−24.2%
Jones et al. [18]	26.9%	$5,400	−19.7	−23.1%

Improved functioning associated with THR is consistent with a 27–28 percentage point increase in employment, $5,400 to $6,200 in higher annual household income, 19–22 fewer missed work days, and a 23–24 percentage point decrease in probability of receiving SSI payments.

To better understand the equivalence of results from the two regressions (i.e., the full model that uses detailed information on patient physical function limitations versus the use of the physical function index), we simulated change in economic outcomes for the NHIS sample under a scenario where patient functional ability improved. The scenario assumed that for each person who found it very difficult or they were unable to walk, climb, or stoop their status improved to only “somewhat” difficult to perform these activities. We simulated the economic results for a population of 185,829 adults over age 18 (all of whom had various levels of functional ability including individuals with no functional limitations). Among this initial population the actual labor force participation rate (unweighted sample) was 62.70 percent. Simulating the improvement in physical function, the participation rate increased slightly to 62.80 percent when using the full regression model and to 62.82 percent when using the index regression. Both models predicted similar small increases in participation rates for the population age 18 to 39 (0.025 percentage point increase from the full versus 0.027 point from the index regressions), and similar small increases for the oldest population (0.079 versus 0.085 point increase). The largest discrepancy in participation rate increase was for the age 55 to 59 population (0.186 versus 0.223 point increase). Both models predicted similar patterns of change in other economic variables (missed work days, disability rates, and household income). In general, we found that the index appears to provide more stable results than does the full model when running alternative scenarios regarding improvement in health. The main reason is that because of small sample size for the NHIS population with certain physical function limitations (e.g., unable to stoop), individual coefficients from the full model sometimes are imprecise (i.e., they have large standard errors and sometimes have the opposite sign than expected). Still, both regression specifications produce similar results.

While many of the patients who receive treatment for MSK conditions are over age 65 (and thus mostly retired), our analysis of publically available data sources (2007–2009 Nationwide Inpatient Sample, and 2009 State Ambulatory Surgery Databases for Colorado, Florida, Maryland, New Jersey, New York, and Wisconsin) suggests a sizable population receiving surgery for MSK-related conditions is still in their prime employment years. Approximately 40% of total knee replacements, 13% of hip-fracture surgeries, 81% of lumbar disc herniation operations, 70% of rotator cuff repairs, and nearly 100% of anterior cruciate ligament reconstructions occur in patients who are under age 65. While the oldest elderly likely will not experience economic benefits from remaining in the workforce, restoring physical functioning through appropriate treatment presumably has other economic benefits such as reducing the need for assisted living or nursing home care.

## Discussion

This study demonstrates the relationship between physical activity limitations and key economic outcomes, which when combined with information from clinical trials produces estimates of the economic impact associated with receiving surgical treatment. Using a large (n=185,829), representative sample of non-institutionalized adults in the U.S., we find that many activity limitations are associated with lower probability of being employed, lower household income even when employed, higher missed work, and higher probability of receiving disability payments. The findings show a relatively consistent dose–response relationship correlating rising levels of physical difficulty with declining economic outcomes.

Although regression results are not reported here, our analysis of the NHIS suggests that some MSK-related difficulties, such as difficulty walking, are associated with higher body mass index and decreased level of physical activity (which raises patient risk for diabetes, cardiovascular disease and other chronic conditions). Likewise, presence of functional limitations appears correlated with increased prevalence of clinical depression (which can exacerbate the burden of MSK disorders). Using a similar regression-based approach to analyze the 2010 Health and Retirement Study (HRS), which asks physical function questions similar to those in the NHIS, we find evidence that MSK-related physical limitations are associated with higher likelihood of hiring of a personal aide and incurring out-of-pocket expenditures for home modification [28].

This study uses published information on patient changes in physical functioning (pre-and-post surgery) as collected through the SF-36 questionnaire. The referenced studies are not randomized clinical trials, with physicians, surgeons, and patients influencing whether the patient receives surgery—even when initially assigned to a non-surgical treatment (control) group. The decision to receive treatment is influenced by the likelihood that the patient will benefit from surgery. Hence, these results may overstate the benefits of surgery on randomly selected patients with equivalent pre-surgery SF-36 scores.

While a large, randomized clinical trial could provide more definitive estimates of the economic implications of MSK disorders and treatment, such an approach presents ethical, financial, and logistical challenges. For many patients and conditions the lack of treatment is not an option. To obtain precise estimates for some measures (e.g., household income) that vary substantially by individual, a large sample size would be needed and information would need to be collected over many years to understand the full economic impact over one’s lifetime.

The focus of this paper is on the relationship between MSK-related activity limitations and employment, household income, missed work days, and disability income; however, there are additional economic burdens imposed by MSK disorders. For example, Ricci et al. conducted a study of 412 U.S. workers with and without clinically meaningful back pain (characterized by frequency of episodes) [29]. The authors report that out of 320 respondents with meaningful symptomatic or controlled back pain; 16.8% reported lost production time, with 79.6% of productivity loss attributed to presenteeism (defined as reduced job performance due to poor health despite being at work). Mean hours lost due to presenteeism for the two-week period were 4.4 hours per worker. MSK disorders can also lead to increased need for long term care [30].

A large portion of the societal economic benefits from receiving appropriate medical care relates to keeping people productively employed in the labor force. Study findings suggest that improved physical mobility can enable more individuals to postpone retirement—which has implications for policies such as raising the eligibility age for social security and Medicare, as well as Bureau of Labor Statistics findings that many older workers have recently been delaying retirement, with this pattern expected to exist even after the economy recovers [31].

Increasingly, policy makers and payers are focusing on value, as evidenced by the Affordable Care Act (ACA) of 2010, which mandates that the Centers for Medicare and Medicaid Services (CMS) test new models of health care delivery in Medicare and Medicaid and implement those that are effective at improving quality and reducing costs. Indirect economic effects constitute a portion of the total value of treatment, and for some conditions and treatments these economic effects are substantial. The findings from this study suggest the potential for large economic benefits from receiving appropriate treatment for MSK disorders, but clearly more research is needed to gain a more complete picture of the value of care.

## Competing interests

This study was completed with funding from the American Academy of Orthopaedic Surgeons, 6300 N. River Road, Rosemont, IL 60018, United States.

## Authors’ contributions

TD helped design the study and drafted the manuscript. PG completed the analysis, modeling, and literature review. LK contributed to the study design and manuscript editing. QG and DR contributed to the literature review and model validation. All authors revised, read and approved the final manuscript.
